# Education of students with intellectual disabilities at Technical Vocational Education and Training institutions in Botswana: Inclusion or exclusion?

**DOI:** 10.4102/ajod.v10i0.790

**Published:** 2021-10-22

**Authors:** Macdelyn Mosalagae, Tanya L. Bekker

**Affiliations:** 1 Department of Studies in Education, Faculty of Humanities, University of the Witwatersrand, Johannesburg, South Africa

**Keywords:** Botswana, capabilities approach, disability, epistemological access, exclusion, formal access, inclusive education, Technical and Vocational Education and Training

## Abstract

**Background:**

Despite a commitment to achieving inclusion for all by the Botswana government, the enrolment of students with disabilities in Botswana’s Technical and Vocational Education and Training (TVET) institutions is still fraught with confusion as reflected by its practice and implementation. Exclusionary rather than inclusive practices remain prevalent.

**Objective:**

This study explores students with mild intellectual disabilities’ experiences of inclusion or exclusion in TVET institutions using key concepts of the Capability Approach.

**Method:**

A phenomenological interpretive qualitative design was adopted. One government, technical vocational institution, offering programmes for students with disabilities in Botswana was conveniently selected. Fourteen students were purposefully selected from this institution based on the criterion that they were students with mild intellectual disabilities. Individual interviews served as the data collection method to enable participants to voice their experiences of inclusion or exclusion at the TVET institution. Thematic content analysis was utilised to analyse the data.

**Results:**

It was found that whilst students with mild intellectual disabilities are offered an opportunity to enrol at TVET institutions, they are faced with social and epistemological exclusion, deliberate marginalisation, labelling and emotional abuses.

**Conclusion:**

These negative experiences hinder students’ achievement by limiting their capabilities.

## Introduction

Current educational policies developed in most countries have a primary mandate of leaving no one behind. Given this mandate, of particular concern is the educational sector’s resourcefulness to meet the needs of all regardless of different educational needs and/or disabilities. There is an increase in the diversity of students across educational levels and particularly in higher education (Svendby 2020). This diversity results in complexities for institutions to adapt to students’ needs especially for students with disabilities. Policymakers and institutional leadership need awareness of the inclusive experience of students with disabilities to evaluate the institutional practice of inclusive education. Inclusive education is ‘an educational philosophy and practice that aims to improve the learning and active participation of all the students in a common educational context’ (Moriña 2017:3). This article investigates the experiences of students with disabilities at a Technical and Vocational Education and Training (TVET) institution in Botswana. The intention is to draw on the students’ experiences to find out in what ways the TVET system has included or excluded them. The students’ experiences presented in this article describe social and academic experiences in relation to inclusion in the institution. The focus is to understand these experiences within a selected TVET institution through the lens of the Capability Approach (Nussbaum 2000; Sen 1999).

Access to education in general and access to vocational education are primary means of enhancing career and employment opportunities for all people including those with disabilities. It is, however, frequently people with disabilities who experience limited access to these opportunities. The opportunities for inclusion of students with disabilities in Israel, the United States and Great Britain are estimated at 8% – 14% (Sachs & Schreuer 2011). Whilst in Africa, 1% of them constitute total enrolment of students with disabilities in higher education (Ngwena et al. 2014). These low percentages indicate challenges of equal participation for students with disabilities in the higher education sector globally (Sachs & Schreuer 2011) as well as in Africa. In Southern Africa, not only are there enrolment challenges but there is also epistemological exclusion because of inaccessible curriculum and inequality in the distribution of resources within higher education institutions. Moreover, a persistent lack of training in disability issues for personnel working with students has been observed by Chakaita (2010) in Zimbabwe, Mutanga (2018) in South Africa and Subbie (2014) in Uganda.

In Botswana, students with disabilities make up 1.9% of the higher education enrolment according to the Botswana Human Resources Council statistics. This statistics indicates the low enrolment number of students with disabilities in higher education (Eide & Mmatli 2015) and does not address the potential concern of epistemological access within the institution once enrolment is obtained. This suggests limited inclusion opportunities for students with disabilities in higher education, which motivated us to find out the experiences of students with disabilities in the TVET sector as far as their inclusion is concerned as a first step to address exclusionary practices. Guided by Sen’s (1992) assertion that all human beings possess agency, it becomes imperative to address these exclusionary experiences because students with disabilities do have agency and aspiration. They also need to be treated like other citizens of the country who have the right to the active participation of what they have reason to be and do (Sen 1992). Furthermore, it is essential to reflect on what students with disabilities encounter in TVET institutions to deepen the understanding of inclusion to combat exclusion in TVET.

## Background

### Inclusive education in Botswana vis-à-vis international policy – How does Botswana understand inclusion?

The understanding of what constitutes inclusion in education has been a global development over time beginning in 1948 with the United Nations (UN) adoption of the Universal Declaration of Human Rights (UDHR). This declaration highlighted social justice, equality and the right to education for all (EFA). The UN Convention on the Rights of the Child in 1989 affirmed the right to EFA children and emphasised that this right should be non-discriminatory. At the Jomtien conference in 1990, EFA was proposed, and equal rights to education for the disabled were emphasised. In 1994 in Salamanca at the World Conference on Special Needs Education, policy revisions for the development of inclusive education were considered. The Salamanca Statement and Framework for Action on Special Needs Education (UNESCO 1994) shifted EFA’s emphasis, and whilst acknowledging equal rights for the disabled, extended focus to acknowledging each child is different and has unique learning needs, and that educational systems and programmes should accommodate for diversity. It was suggested that learners with barriers should be accommodated in mainstream school settings, and EFA achieved through schools adopting an inclusive orientation (UNESCO 1994). Moreover, the Salamanca Statement affirmed the need to combat exclusionary and discriminatory practices and change societal attitudes towards difference. The EFA goals were reiterated at the World Education Forum in Dakar in 2000, where they were endorsed and adopted and where the needs of the poor and disadvantaged were included as important for consideration in acknowledging diversity. These international developments demonstrate a shift from an initial focus on special educational needs and disability primarily to a broader consideration of recognising difference and diversity and combatting exclusionary pressures and practices as a key priority for inclusion in education.

Alongside international developments clarifying and elaborating on what inclusion in education means, individual countries were simultaneously developing their own educational, legislative and policy responses to developing inclusive education systems within their unique contexts.

Miles and Singal (2010) suggest that inclusive education needs to be considered in relation to specific cultures and contexts to attend to educational inequalities specific to those contexts. This suggests that whilst inclusive education is a global common principle, it cannot be practised in uniformity. Although there is a consensus on the importance of inclusive education, its interpretation and the programme of action will always differ (European Agency for Special Needs and Inclusive Education 2017). We argue that the understanding of inclusion cannot be comprehended by disconnecting from other situations around the world as challenges to the actualisation of inclusivity are universal (Armstrong, Armstrong & Spandagou 2010; Walton 2015) whilst acknowledging different contextual realities. Therefore, schools and governments should look at incidents in communities that perpetuate inequalities in order to understand features that promote exclusion (Slee 2001). With this understanding, it is notable to explore the specific context of Botswana.

Despite Inclusive Education shifts from a specific focus on special education to a broader focus that recognises disability as one aspect of diversity internationally, Botswana still tends to operate from a standpoint of special needs. Hence, Botswana continues to establish special education units for students with disabilities in both rural and urban areas of the country (Dart 2007; Molosiwa & Mpofu 2017). The most recent and first of its kind being the establishment of a Special Needs department at one of the TVET institutions to cater for students with mild intellectual disabilities. With this practice, Botswana has neglected to recognise the broader conceptualisation of inclusive education and its subsequent reconsiderations as a unified education system. This failure also contradicts the conventional Botswana Inclusive Education Policy, which states that:

[*A*]n Inclusive education system is when special educational needs of young people and adults are met in mainstream pre-schools, primary and secondary schools, vocational training programmes, colleges and universities with appropriate teaching and support. (GOB 2011:4)

The existence of the Inclusive Education policy proves that there is a common understanding from policymakers in Botswana, but that this is not necessarily being implemented in practice. The existing implementation demonstrates how Botswana, despite the introduction of an Inclusive Education policy in 2011, has not shifted from the traditional view that the education of children with disabilities is only possible in segregation. We are of the opinion that given where Botswana is operating from, inclusive education in Botswana vis-a-vis the goal of international policy on inclusive education has remained a policy statement and has failed the expectations of the principles of inclusive practices (Muzata et al. 2019). This challenge is not only to Botswana but also a challenge faced in most of the Southern African countries (Pather & Nxumalo 2013) that are failing to address barriers in the system that restrict the education of marginalised individuals including children with disabilities. The key challenge is the lack of understanding that inclusion is a flexible way to allow improved support in the education system, and it is not about merely identifying learners with special educational needs who are deemed to be educated separately.

In terms of developments in the TVET sector, Botswana like other African countries was compelled to respond to the global policies such as Salamanca Statement and Framework for Action on Special Needs Education of 1994, even though the progress has been challenging (UNESCO 2015). The inclusion of marginalised communities and people in Botswana follows the global pattern of action in response to Development Goal No. 4 and the EFA (UN 2015). Sustainable Development Goal 4 speaks of ensuring inclusive education and equitable quality education as well as opportunities for all (UN 2015). This goal aims at reducing barriers to skills development in TVET education (UN 2015). In line with this goal, Botswana is reported to have taken a stride to ensure the right to education of people with disabilities through its longstanding National Policy on TVET of 1997 (Mmolai 2019; Ndzinge-Makhamisa 2019), which originated before the SDGs. The vision of the policy is to provide access to vocational training with disadvantaged groups as a priority, and this stance tallies with Botswana’s action measures on the implementation of inclusive education.

Botswana’s articulation of inclusive education refers to ‘an education system that includes and meets the needs of all, including those with special educational needs, those with life circumstances, health, stages of development or any other circumstances’ (GOB 2011:4). One could understand Botswana’s articulation of inclusive education and inclusion to imply that the society has obligations to take care of each of its members regardless of mental, physical, behavioural and emotional status. If Botswana understands inclusion from a societal obligation standpoint, it is necessary to consider in more detail the policies that support this stand.

### Policies guiding Technical and Vocational Education and Training in Botswana

Botswana’s first policy on education entitled Education for Kagisano of 1977, the subsequent Government Paper No 2 of 1994 and the National Revised Policy on Education (RNPE) have been instrumental in leading Botswana’s education system in an inclusive direction. The policy recognised that Botswana’s breakthrough to economic diversification is through VET. The RNPE advises that whilst the industry commits to specialised vocational education, the government should be in charge of primary vocational education. This initiative was viewed as the means to achieve economic diversification and become instrumental in addressing issues of theory and practice mismatch (Pheko & Molefhe 2017). The need for vocational education and training (VET) in Botswana was impelled by the country’s economic history (Mupimpila & Narayana 2009; Siphambe 2009), where vocational education was not viewed as a priority and educating for white-collar jobs (workforce in offices). Educating for white-collar jobs meant a focus on theoretical rather than practical courses in many cases. In terms of inclusion, three policies promote inclusion and inclusive education in TVET. Firstly, the VET Policy of 1997, which calls for ‘increase in access to VET by making education inclusive and equitable whilst addressing issues of quality and cost-efficiency’. The second policy is the RNPE, which has a special provision for an education, which caters for children and young people including those with disabilities (Republic of Botswana 1994). The third is the Inclusive Education Policy of 2011, which has 10 goals of which goal no 4 is significant to this article. It states that ‘action will be taken to improve vocational training for young people for whom the current system of vocational training is unsuitable’ (GOB 2011:1). The adoption of these three policies did not, however, mean that these vulnerable groups would access education in practice. The enrolment of individuals with disabilities in TVET is in fact still minimal (EFA Country Profile 2015).

Botswana’s educational development is interpreted with the National Development Plan (NDP), which usually run for 6 years. National development plans in Botswana are reflective frameworks in which government checks signs of progress in the different sectors of the economy. Therefore, it is not surprising that the objectives of the initiative of vocational education continue to change. The study informing this article took place within the NDP 11 plan. The NDP 11 plan commits to the following:

- improving human capital development
- improvements in the quality of education to increase the pool of skills in areas that have been identified as critical to improve the performance of the economy and
- and to ensure that all population groups in the country benefit from an inclusive education and training system.

Whilst the initial vision of TVET in Botswana was for productivism, the reflection from the current National Development Plan (NDP) indicates that the development of human capital is significant to the economy. Hence, the current status of vocational education is not solely government domain, as the growth of private vocational institutions is on the increase (Richardson 2013; Samboma 2017). Not only is the current NDP objective to promote human development but embraces inclusion through inclusive education directives with the emphasis that everybody who has the right to education should be given the opportunity. The NDP 11 provides for the expansion of brigades and technical colleges (Richardson 2013). Brigades are community-led vocational schools, whereas technical colleges are government-led institutions of technical and vocational training. Another government initiative that promoted an increase in formal access for people with disabilities was to earmark certain brigades and technical colleges to offer different programmes for different disabilities. This initiative enabled the admission of the first cohort of students with mild intellectual disabilities in 2012 to the technical college selected for this study. Although this can be viewed as a positive step, the reality of the implementation of inclusive education on the ground seems to be rhetoric. There is still inequality and exclusion of marginalised students in Botswana’s higher education (Makwinja 2020). This supports the consideration of how TVET institutions practise inclusion.

### The practice of Inclusive Education in Technical and Vocational Education and Training

Admission of students with disabilities in the technical college selected for this study follows a process of interviews with candidates and their parents to discuss the educational psychologists’ report, which is a prerequisite for admission. Suitable prospective students are then selected based on current ability levels and the perceived fit with the programme. There is, therefore, accommodation and modification in terms of the admission processes. The college initiative follows the country’s Affirmative Action Framework (Ministry of Education and Skills Development [MoEDS] 2013), which has the goal to ensure that no one is left behind by admitting students regardless of test scores. Current practice in the TVET college is to separate students with disabilities from the so-called ‘normal’ students so that classes for students with disabilities are offered separately. The separate provision is justified as allowing students with disabilities to learn at their own pace in order to have epistemological access. A pragmatic justification for separate classes is that students with disabilities doing hospitality operations have a separate kitchen where equipment has been modified. For example, stove handles have been lowered for any students in wheelchairs. Some classes, however, are still on the second floor of a double storey building making them inaccessible to some, especially when the lifts are not working. Whilst the college attempts to offer students with disabilities the opportunity of learning on their own, it simultaneously portrays a negative view of the students’ disabilities. On a different note, the college’s justification of this separation is that programmes for students with disabilities are below the level of what is offered in the same hospitality operations at diploma level for students without disabilities. This segregation has resulted in labels and othering of students in the department. At the same time, the label is seen by the management and policymakers in the ministry as a means to identify the educational needs of students with disabilities. What the college is experiencing resonates with the ‘dilemma of difference’. The dilemma of difference as coined by Terzi (2005) and Norwich (2010) is a choice between treating all students as the same so that no one feels different and provides similar support and resources or whether to label and treat students differently so that they can benefit from individualised support. Besides the concern for students learning, the college is also faced with the operational mandate. That is, whether the department should be a service department in which it serves the educational needs of all students in the college or as a special unit focusing on the students in their section only.

When students have completed a unit, the student’s disabilities are catered for through modification and enrichment of assessment. Assessment is modified for those students experiencing difficulty and enriched assessment is provided to those for whom the materials are found to be too easy. This speaks of an inclusive assessment practice in which there is a ‘need to review and modify test items to ensure that they have maximum accessibility, without changing the properties of the test items’ (Elliott, Frey & Davies 2015:2). More importantly, the assessment criteria determine whether students with disabilities have achieved the unit independently, with assistance or not achieved at all. This allows these students to be re-assessed until they can achieve. This is also part of an inclusive practice in which students are assessed for checking skills acquisition rather than grading so that students are not left behind.

Initially, students with disabilities at the TVET College were also socially separated from others as their hostel accommodation was separate from the rest of the student community. However, the current situation is that students with disabilities are paired with students without disabilities to promote social integration. Positive results of the initiative include integrated sports where teams were formed and ultimately improved their sense of belonging. Pairing students with disabilities with students without disabilities may, however, be reinforcing difference, labelling and othering. According to Norwich (2010), there is a dilemma of identification. This dilemma means that the students may not be able to enhance their social skills because they are treated by the so-called normal differently but if they are identified according to the disability, it may help the college to secure what will benefit them. For example, the hostel doors were modified to allow wheelchair users. In practice therefore, it becomes clear that at the selected TVET College, formal access to the institution has been facilitated for students with disabilities, but the question of whether full inclusion beyond formal access is being achieved remains.

## Literature review

Scholars in inclusive education have investigated the experience of teachers in implementing inclusion of students with disabilities in higher education as well as students’ experiences in higher education (Kendall 2016; Mutanga 2018; Svendby 2020). It has become clear that the inclusion of students with disabilities is still a challenge as it continues to fail to afford students equal opportunities in learning. For example, Svendby’s (2020) study reveals that teachers lack awareness of students’ disabilities and inclusive pedagogies in their teaching. This lack of awareness suggests that students have not been supported enough to reach their potential. If that is the case, it indicates that students with disabilities have been excluded from full participation in the learning. Dolmage (2017) also observed that disabled students in higher education tend to be excluded from participation because of the assumptions of their ability. Ableist assumptions tend to mark critical disabilities studies where individuals have the tendency of thinking and assuming the worst of students with disabilities. These assumptions come in the form of pigeonholing, stigmatisation and labelling (Boyle & Sharma 2015; Svendby 2020).

International literature has explored the inclusion of students with disabilities in higher education, for example in Malaysia; Yusof et al. (2020) express students’ voices on how they are not on par with their non-disabled students as far as learning is concerned. In the United Kingdom, Kendall’s (2016) study on the experiences of students in higher education reveals that whilst the number of students with disabilities continues to increase in universities, a number of issues that hinder their full participation still occur. In Italy, in exploring the disability and Italian experiences of inclusion, Maggiolini and Molteni (2013) observe that the focus of good inclusive practice does not lie on the students’ disabilities but on how institutions process inclusionary practices. It emerges from this research that underrepresentation of students with disabilities at higher education has improved (Kendall 2016; Maggiolini & Molteni 2013; Yusof et al. 2020) but the path to inclusion still needs further research.

Within Southern Africa, there is literature on the experiences of students in higher education (Matshedisho 2010; Mutanga 2015; Mutanga & Walker 2017; Ndlovu & Walton 2016). For example, Matshidisho (2010:730) argues that ‘even though the experiences of disabled students have programmatic implications, their needs should not be isolated from other students’. We concur and reason that research focusing on one aspect of their life is detrimental to findings on the voices of their actual experiences in terms of inclusive practice. To show this emerging picture, studies performed in South Africa indicate that there are numerous barriers still experienced particularly by students with disabilities resulting in limited professional skills amongst individuals with disabilities (Ndlovu & Walton 2016). Mutanga (2018) also observed that instances of exclusion in two universities in South Africa are apparent, for example, limitation in participation in academic programs of students with visual impairment and challenges of physical access to infrastructure. This demonstrates that challenges still exist despite new legislation and law governing the inclusion of students with disabilities.

In terms of Botswana, literature on published studies on experiences of students with disabilities at higher education seems to be limited. The synthesis of literature points to research focusing on one aspect of students with disabilities, for example, attitudes or perceptions (Mokhuphadyay 2015; Molosiwa & Mpofu 2017; Otukile – Mongwaketse 2011). None of these studies, however, focused on higher education particularly TVET. The same has been observed in South Africa by Mutanga (2017) when he opined that ‘there is scant literature on the experiences of students with disabilities in South African Higher education compared with other countries such as Australia, the UK and the USA’ (p. 136). In the case of Botswana, programmes for access to higher education of students with disabilities, especially in TVET, began in 2012 (Mosalagae 2021), so literature exploring the experiences of the student would invariably be limited. Hence, this article will contribute to the limited literature in this area.

## The problem statement

Studies focused on the TVET sector carried out in Kenya by Malle (2016), Malle, Pirttimaa and Saloviita (2015), who investigated prevailing challenges and opportunities for the participation of students with disabilities in vocational education whilst Murgor, Changa and Keter (2014) in Ethiopia explored the accessibility of TVET amongst disabled people and reported barriers to the full participation of students with disabilities in TVET but did not explore issues of student experiences. Some studies on inclusive education and higher education by Mutanga (2015) in South Africa and Subbie (2014) in Uganda focused on disabled students’ disabilities and experiences in higher education. In Botswana, there are few studies performed in the area of inclusive education in higher education and TVET. There has been little research that explores students’ well-being and experiences. Consequently, very little is known about how students with disabilities have experienced TVET education. These are the impelling motivations behind this study.

## Purpose of the study

Given that there is little empirical research on the experiences of students with disabilities in TVET education in Botswana, this study aims to develop an empirical understanding of the experiences of students with disabilities in TVET education. The purpose of developing an understanding of these experiences to determine whether inclusion in these TVET programmes has been beneficial to these students’ well-being and functioning. The Capability Approach is used as a theoretical framework to explore students’ experiences. We intend to build on current debates on the experiences of the inclusion of students with disabilities and contribute to the literature in this area.

The following research question was posed:

- What are students with mild intellectual disabilities’ experiences of inclusion in the selected TVET institution in Botswana?

## Theoretical framework

Given the understanding of Botswana’s conceptualisation of inclusion and inclusive education, which is the obligation of the society to take care of its members regardless of mental, physical, behavioural and emotional status, the Capability Approach was chosen to evaluate and assess the students’ well-being and how the social arrangements, resources and teaching practices have shaped their experiences of the TVET institution. The Capability Approach was used as a framework to help in conceptualising the students’ experiences and evaluate their well-being. The Capabilities Approach, pioneered by Amartya Sen and developed by Martha Nussbaum, is defined as a normative framework of well-being, human development and justice using functionings and capabilities (Nussbaum 2000; Sen 1992, 1997) and is considered not as a theory but as a moral approach (Sen 1985). For the purposes of this study as a moral framework, it was suitable because in its evaluation it considers information out of the normative (Robeyns 2005). That is, it captures non-utility information. For example, in this article, it considered the intellectual abilities of students, the moral and social issues, such as the humanitarian principle of appreciating them as persons first before their disabilities. The capability approach then endeavours to focus on how well a person is and what the person does to attain that wellness. Wellness, in this regard, denotes the manifestation of what a person can do (functionings) with what he has (Sen 1985). As a result, the approach does not cause so much concern itself with the possession of resources by an individual or the state (mental) that a person is in, but rather the freedom the individual has to arrive at what he or she has reason to value (Sen 1985, 1992, 1999). Nussbaum (2011) attests to the fact that the Capabilities Approach has two claims. Firstly, the chief moral aim is freedom to achieve well-being. Secondly, the very same freedom towards the achievement of well-being is to be appreciated in relation to the capabilities of people, that is, their actual chances to do and be what they have reason to value (Nussbaum 2011). The expansion of human capabilities helps one to have agency, that is, acting and bringing changes to one’s life. Central to the Capability Approach is the enhancement of capabilities; therefore the answer to finding out if inclusion in TVET is being fully realised lies in whether it was able to develop capabilities expected from higher education. The Capabilities Approach asserts that students with disabilities are capable beings (Davis 2006; Oliver 1996; Shakespeare 2010), especially when they are given supportive and inclusive environments. Sen (1999, 2000) argues that appropriate provision affords agency and enhanced capability. Employing the Capability Approach was relevant as an analytical tool to provide a ‘mirror’ for reflecting on the benefits of TVET programmes in assisting students with disabilities to achieve well-being. Based on student experiences, we were also interested in finding out practical ways in which TVET can expand the capabilities of students by considering the social arrangements (the institution, programmes, stakeholders and policies) to find out inclusive opportunities and unfreedom instances that were regarded as exclusions. The Capability Approach emphasises the evaluation of capabilities and functionings. In this article, we looked at relevant capabilities including educational resilience, social relation and social network, respect, dignity and recognition, knowledge and imagination and practical reasoning. We then indicated how they are related to experiences of inclusionary or exclusionary practices. For example, the experience of being loved, care for by others and a sense of belonging are experiences of capability of affiliation and that speaks to social inclusion.

## Research methodology

The study used a qualitative research design to investigate the experiences of inclusion of students with mild intellectual disabilities at one TVET college in Botswana. The study used an interpretive phenomenological approach in which the ontology was to socially construct the participants’ meaning (Male 2016; Maxwell 2013). Interpretivism was thus ideal for this study because it concerned itself with understanding the world as it is from the subjective experiences of individuals. This suggests that we sought to understand people in their role as social actors and that the original description of what inclusion meant from the participant’s view was captured verbatim without any alteration. Adopting this stance for this study is supported by Male (2016:125) who asserts that interpretivism is a ‘conceptualization process of how situations are meaningfully lived as they are experienced with nothing added or subtracted’. This shows that in the field of social sciences, truth is not final. The Interpretivist Design was appropriate for this study given the intention to uncover new thoughts, gain new understanding as well as increase knowledge of inclusive education.

### Sampling

One TVET college in Botswana was selected as the site for this study using convenience sampling as it is one of the two institutions that offer programmes for students with disabilities. The participants were purposefully selected for the study to get relevant information for the study. Purposive sampling is a subjective non-probability sampling in which participants are chosen through the judgement of the researcher (Patton 2014). The experiences of students could not be attained from people other than the students themselves; hence, purposive sampling was relevant. Participants were 14 students with mild intellectual disabilities who were enrolled in the Hospitality program. These students were chosen on the criterion that they are students with mild intellectual disabilities and as the technical college admits this specific disability. A characteristic list was made to further sample participants based on the ability to understand both Setswana and English, ability to communicate well, the chronological age of 18–23 years and programmes of study (in this case, Hospitality Operations). Ethical clearance was obtained for the study from the affiliated higher education institution, and all procedural ethical processes were followed, including obtaining informed consent from all participants, ensuring anonymity and maintaining confidentiality. The following data collection tool was used to construct participants’ meaning of their experiences of inclusion at the selected site.

### In-depth interviews

In-depth individual interviews were conducted with both the currently enrolled and graduated students’ participants. The interview was guided by a semi-structured interview schedule where questions focused on the participants’ experiences of inclusion and exclusion in the TVET college. The individual interviews were critical in having a ‘conversation with a purpose’ with participants. A semi-structured approach was adopted to ensure attention to critical key questions related to students’ experience of inclusion but also to allow for probing and elaboration of these responses. Considering the realities that student meanings of experiences are different and unique to individuals, we found it essential to use individual interviews so that we could probe further and clarify participants’ meanings so as not to misinterpret their real experiences (Rubin & Rubin 2012). Fourteen individual interviews with currently enrolled students were conducted. The interviews were employed to contextualise their meaning through their voices, and therefore they served as a space for understanding those voices (Creswell 2014). These voices were audio-recorded verbatim and later transcribed.

### Data analysis

The study’s data analysis was informed by its epistemological position, which is to interpret meanings. We employed an iterative process of data analysis. An iterative process means that we had to look at the data as a whole through its parts and vice versa to ultimately come to a context in which the whole and the parts were embedded (Smith, Flowers & Larkin 2013). With this process, we had to keep an open view of what the implication of the data might mean. For instance, data items were analysed individually looking at what students said, what it meant for each data set and ultimately what the interpretations were. This progression aided us to arrive at what Holloway and Todres cited in Braun and Clarke (2006) call ‘thematising meanings’. In the words of Braun and Clarke (2006:6), ‘thematic analysis is a method of identifying, analysing and reporting patterns (themes) within data’. Prior to thematising, the following process was ensured.

Recordings from the individual interviews were translated verbatim from the vernacular to English, and the English versions were then transcribed. Particular attention was paid to the individuals’ contextual experiences. Engaging with data assisted us in extracting and identifying significant meanings as told by the participants. Transcripts were manually coded following three steps. Step one was to open the code drawing from the literature review to identify essential elements that could possibly make a pattern by comparing and categorising (Saldaña 2013). The second step was the axial coding, looking at the causal relationships (Saldaña 2013). In this step, a sequence of thematic codes was generated by employing the Capabilities Approach (affiliation, agency, freedom, capabilities and functioning). This follows Neuman’s (2006) idea that at this stage a clear link between the concrete findings and the theoretical themes should be noticeable. Thematic analysis was not limited to original themes but was flexible to patterns that came out and were helpful to answer the research question beyond the initial themes. The third step was selective coding, which helped to develop explanations and move beyond the descriptive analysis of the data. We looked for themes through the thematic latent analysis level. The latent analysis level demands that a researcher goes beyond the surface meaning but reaches themes through interpretations of the data by identifying features and ideologies that go beyond the meaning (Braun & Clarke 2006). For instance, in this study, we analysed interview extracts by not only looking at what the literature refers to as elements of inclusion but we also used information related to the research question and its relation to the Capability Approach.

### Ethical considerations

Ethics clearance was obtained for the study from the University of the Witwatersrand, reference number: 2017ECE017D.

## Results

The results presented speak to the research question that sought the experiences of students with disabilities of inclusion and exclusion in TVET. The presentation of findings and discussion that follows considers experiences of inclusion and exclusion as grouped and classified from the interpretations of student’s experiences during thematic content analysis. Table 1 shows how themes were arrived at by not just a description but also by theorisation after linking the research question with the theoretical framework. The presentation of findings is presented as two overarching themes, namely: (1) Experiences of Inclusion in TVET and (2) Experiences of Exclusion in TVET.

**TABLE 1:** Examples of how themes were created.

Research question	Interview extract	Theoretical framework descriptors	Relational to inclusivity
**What was your relationship with others (classmates, other students and staff?)**	‘We have lecturers who were from other departments who are good and work well with us, and then there are special needs lecturers who are okay, only that there was one who takes herself as the “the person” and other lecturers were afraid of her even to tell her that she is not treating us well. So, she could not even see that she is not treating other people well. Then, there are my classmates who are afraid of their lecturer. And she put their morale down. Personally, I do not have any relationship with her, even when I have a problem, I would not tell her. I prefer to tell other special needs lecturers.’	Being able to work with others/cooperation Not being able to develop emotions for understanding and awareness Fear that diminishes learning and subjected to anxieties Unable to form a network of friendship for effective support and mutual trust	Social relationships (social inclusion) Communal practice (Botho) Workmates and students bully. Victimisation (social marginalisation and exclusion) Emotions and demotivated (epistemological exclusion) No sense of belonging. (Social exclusion)

### Experiences of inclusion in Technical and Vocational Education and Training

**FIGURE 1:**
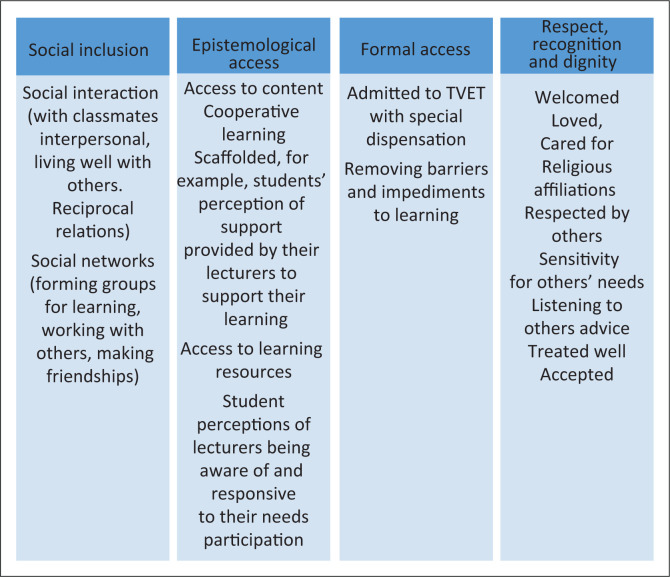
Inclusion and inclusion indicators.

The experiences of students in relation to inclusion are summarised in Figure 1, which shows indicators of how inclusionary practices were identified from the data. Four key areas were identified, namely (1) social inclusion; (2) epistemological access; (3) formal access and (4) respect, recognition and dignity.

The analysis showed that students experience some level of good inclusionary practices by being socially included. Students reported having been able to interact with others socially and form friendships. The interpersonal skill modules helped them to live well with others and have reciprocal relations with some lecturers and the college non-academic staff.

Being enrolled in TVET gave the students epistemological access. Here, students were given the opportunity of gaining new knowledge through accessing learning resources and lecturers knowing their profiles that helped with being scaffolded and remediated where necessary. The students also benefited from the integrated teaching that lecturers employed. Integrated teaching between the vocational lecturers and the special educators enabled the students to be given attention and units objectives tailored to their profiles. In this way, students were able to access content that eventually gave them some capabilities achievement. New knowledge acquired shaped students’ aspirations for their future. Box 1 demonstrates an example of these aspirations as expressed by student participants.

BOX 1 Student’s aspirations.

**Nox:** ‘When I started in this college, I was from a special unit class and I had no idea of what happens in tertiary schools. As I attended here, I have learnt a lot of new things from the hospitality course, and I love it here because I now have new knowledge. I now know how to deal with customers, cook and housekeeping. I never knew that one day, I will also know new things. What I want to do is that I want to cater to people like in weddings and open my own business, I would want to negotiate with companies to assist me and look for a plot where I can operate from.’

**Precious:** ‘I am happy that I came to this college, I was just at home and doing nothing, now I have new skills like using a fax machine, laminating, photocopying, etc. I have learnt entrepreneurship from this college. So, I will start my small business and be self-employed. I want to open an internet café.’

Some students were also grateful for the TVET experience because they felt a sense of belonging. They were able to live well and interact with others. This speaks to the capability of affiliation (Nussbaum 2000). The achievement of this capability meant that these students were able to form social networks, which were instrumental in their forming learning groups, which helped with other capability achievements such as numeracy, literacy, ICT and problem-solving skills to name a few. Also being able to relate well resulted in students being able to accept their disabilities, eventually gaining confidence and self-esteem as well as being able to take risks without fear.

Social inclusion not only benefited students in forming networks for learning, it made a number of students feel welcomed and cared for by other non-disabled students and other peers with disabilities. Ultimately, students could be recognised, respected and feel dignified. The feeling of being accepted was instrumental in students being able to appreciate TVET as it translated to formal access. Formal access in the sense that nearly all students felt that the impediments such as not being accepted and treated well would have hindered their learning experience. Therefore, barriers that impeded learning were removed. Removal of barriers also meant that a good number of students were allowed to enrol in TVET, especially when special dispensations were made so that they may qualify to attend TVET. One of the dispensations was that students were not subjected to entrance examinations. Box 2 demonstrates examples of these experiences as expressed by participants.

BOX 2 Students’ experiences of inclusion.

**Mpule:** ‘After completing junior school, I had lost hope that I will never be anything in life. But the experiences I had at college taught me a lot. I have grown a lot and even when leaving college, I realised that I have changed. I learnt to live with people. I have been able to participate in things I did not like, so I realised that I have the potentials. That is why I was able to go to America because of my potential.’

**Patty:** ‘In this class, we had bonded well and even those who used the wheelchair, we had taken them as people who can walk, even when we went to America were not afraid to tell people that we have disabilities.’

### Experiences of exclusion in Technical and Vocational Education and Training

**FIGURE 2:**
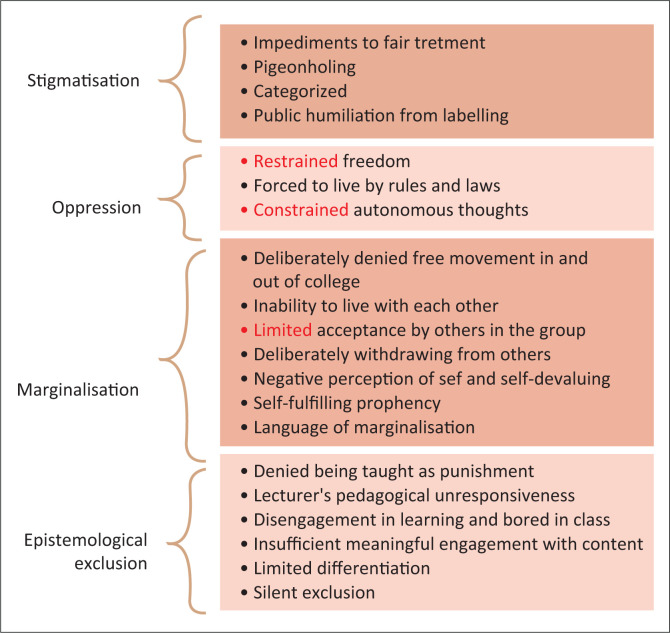
Exclusion and the indicators.

Whilst some students experienced the above-said inclusionary practices, many also experienced forms of exclusionary practice. This is not surprising as literature reviews indicate that where there is inclusion, there is also exclusion (Ainscow, Booth & Dyson 2006). Figure 2 demonstrates exclusionary practices and its descriptors as identified from data analysis.

At times some students felt epistemologically excluded when certain lecturers failed to help them engage with content. For example, some students were afraid to tell their lecturers when they had a problem with content because certain lecturers were impatient with students. Some lecturers used abusive language, which resulted in students not wanting to come to class or feeling intimidated. In such instances, affected students revealed that they resorted to not asking questions and not participating in class. Box 3 captures student expressions of these experiences.

BOX 3 Student’s experiences of exclusion.

**Kabo:** ‘I don’t have any worst experience except for my class lecturer who always uses abusive language in reference to us. I find it impossible to live with her and I don’t like her. When I have to attend her class, I am not happy. She is moody and she brings her stress from home to class so I always feel like staying away from her class, just stay in the hostel. I am not happy at all with her. I don’t have a relationship with her.’

**Snowy:** ‘Coming to lecturers, one of them was not free, Jealous and discriminatory with us, and I was not comfortable with her. Every time I had to go to her class, I felt stressed and did not like to go. It’s like she was not comfortable with teaching us. I knew that if I raised my hand, I will not be given the chance to speak So I ended up not raising my hand in class. And even when I wanted to ask something, I was afraid, and that made me not to have a relationship with her because I was afraid of her.’

Some students reported that one particular lecturer used to suspend teaching them as a punishment when she found fault with them. They experienced this as an unfair punishment measure. Students believed that this suspended teaching was used for them because they were ‘special needs’ students. Students cited incidents where they were punished for not knowing that there was a class by coming to class and then not being taught. At times, students also felt silently excluded because they were in class and college and yet not epistemologically benefitting. Silent exclusion (Lewin 2009) refers to enrolling students in schools, yet little learning is taking place in the classroom. This results in constrained engagement with the curriculum as students are physically present but not supported to access knowledge (Walton & Bekker 2013).

Another exclusionary practice experienced by students was marginalisation. Data showed that students were denied free movement in the college as all participants reported that they were restricted to the hostels only and particularly barred from making friends with students without disabilities. Whilst this restriction occurred in the college, it was, however, a practice of a particular member of the department. She threatened students without disability that they would be taken to task if they befriended students with disabilities because it was ‘not allowed’. In contrast, students with disabilities were threatened that they would be expelled from college if they were seen with non-disabled students. The fear that was instilled in students resulted in them keeping to their hostels. From a Capability Approach perspective, this kind of practice meant that students were denied the capability to take control of their environments (Nussbaum 2000) as they had no freedom of association.

They were also not allowed to go outside of college independently. Students were only allowed to leave campus with the permission of their parents who then needed to come and collect them from college. This practice was not welcomed by students who felt that they were caged, and their independence was taken away from them. This is also contradictory to what they are being taught in curriculum regarding independent living. At the same time, students felt that they existed between two personas as, when they are outside college, the society treated them as abled persons whilst in college where they are supposed to grow, they were treated as ‘babies’ or ‘little mice’. Box 4 provides examples of students’ expression of these experiences.

BOX 4 Student’s experience of labelling.

**Mantsho:** ‘I once wanted to leave this course because when I am outside of college, people treat me normally and I function well, but when I get to college, I am treated like I am baby who has limited thinking. I am always told that I am limited and I am a “special needs”.’

**Gomo:** ‘I am different in a particular way, just that we have mild intellectual disability does not mean that we are different. People segregated us and take us lightly. I think they take us lightly because we are in special needs department and therefore takes us for granted.’

Besides being deliberately restrained by the department rules, some students also reported the use of language of marginalisation. They were called by names by some lecturers and other students without disabilities. These name callings affected the students’ self-esteem as they reported that they saw themselves as those names and ended up believing that they are indeed what those names mean. For example, being called ‘special need’ meant that they are exceptionally not normal hence ‘special’. According to McDaid (2008), often people with disabilities are perceived in terms of the challenges they have without considering their overall contribution in the society and many times they are understood as dependent on others as well as incompetent. Labelling or not labelling in the field of special education is debatable. However, the Special Educational needs and Disability Act of 2001 (SENDA) and the Special education law of 2004, which was renamed the Individual with Disabilities Education Act (IDEA), allow labelling if they are meant to categorise an individual so as to address their educational needs for their individualised learning programs (Boyle 2014; Boyle & Sharma 2015). On the contrary, given the stigma generally allied with labelling individuals, it has been argued that labelling usually ignores the need of the individual and focuses on pathological tendencies (Goodley 2001). This suggests that labelling or categorisation is supported in legislation, where the intention is the provisioning of support, but that the practice of doing so may result in the experience of stigma for individuals so labelled.

Whilst labelling in inclusive education is not something new (Boyle 2014; Boyle & Sharma 2015), we argue that the policy landscape and school system socially constructed the labels, which the collegiate community adopted and used to refer to these students. In other words, the use of the word ‘special needs’ became an institutional practice, which was exclusionary in nature. Becker’s (1963) labelling theory suggests that labels serve as special markers, which are used to identify and classify particular students in institutions or society. Although labelling to other scholars (Booth & Ainscow 1998) is to recognise differences and confer social salience to those differences (Arishi et al. 2017), in the context of this study, some students experienced labelling as a discriminatory practice despite it being used by the department for the purpose of special educational provision. Studies have shown that marginalisation goes hand in hand with stigmatisation. A study by McDaid (2008) showed that professionals are also amongst the populations that stigmatise within their profession. This was the case with this study, where students were stigmatised by their lecturer who dehumanised them verbally, physically and emotionally. Evidence of this is provided in student statements in Box 5.

BOX 5 Student’s experience of marginalisation and stigmatisation.

**Bame:** ‘I don’t have any challenge academically but the challenge I have is the lecturer who teach me. She does not allow me to be creative, for instance, if she gives us homework and ask us to give two examples if I give more than that she will shout at me and tell me that my mind is limited to the number of examples she said I should give.’

**Nkabi:** ‘I have a relationship with other special needs lecturers except the one who calls us by names and tells us we are limited and that is why we belong to the special needs department. I hated her and always wish I don’t come to her classes.’

Some students were labelled and deemed incompetent. One of their lecturers communicated low expectations of them instead of having a positive attitude, which could have countered collegiate community negativity. The stereotyping that some students experienced resulted in them being categorised and pigeonholed. This labelling increased the distance between provisions that students were afforded. For instance, some students without disabilities treated students with disabilities as not equal to them. The behaviour yielded sexual abuses with the belief that students with disabilities would not be able to reason and identify their perpetrators. Not only was a student with disabilities sexually abused, but some students with disabilities were also taken advantage of in terms of their possessions. Furthermore, outside people masquerading as churches and insurance people wanted to rob them of their allowances. Box 6 shows some of the excerpts that demonstrate these experiences.

BOX 6 Student’s experience of consequences of negative stereotyping.

**Tally:** ‘There was once a church that came to school and tried to swindle us some money. They told us to contribute P100 every month so that when we fail in GTC because we are special needs student, they give us the money. [*Some*] people think because we are in special needs, we do not think and they can just do what they want with us. We have disabilities but we are able[*-bodied*] individuals.’

**Ndongo:** ‘One boy once came to my room and locked me and said I slept with him. When I told him I was having my period, he said I should suck him.’

Negative experiences such as these hindered students’ well-being achievement by limiting their opportunities for expansion of capabilities. It becomes clear that both inclusionary and exclusionary practices were experienced by students with disabilities in TVET. Of the two practices, exclusionary practices were noted more than inclusionary practices in terms of references made by students during interviews. This suggests that TVET is struggling with the implementation of inclusive education suggesting that inclusive policy is not fully realised in practice.

## Discussion

The result of the study reveals that both inclusionary and exclusionary practices were simultaneously experienced by students with disabilities in TVET. This supports Sayed, Soudien and Carrim’s (2003) assertion that inclusion and exclusion are two concepts that are not competing paradigms because of their connection. Data show that the experiences of students were as unique as their own differences as individuals.

Some students experienced inclusionary practices as shown by data examples of living harmoniously with other non-disabled students and the collegiate community; forming friendships and teamwork in learning, gaining new knowledge and skills from the course they were pursuing; new dreams for their future because they have been empowered and skilled as a result of good teaching methods, good resources and a conducive environment of learning. We are of the view that such results imply that from a lens of inclusionary practices, students experienced epistemological access and social inclusion. The contribution of the Capabilities Approach to this result indicates that TVET had given students opportunities to learn. In the process, students’ acquired capabilities of affiliation Nussbaum (2000); or social relations and social networks (Terzi 2007; Walker 2006); sense, imagination and thoughts (Nussbaum 2006) and practical reasoning (Terzi 2007; Walker 2006) were also gained capabilities. Therefore, in terms of TVET education, the capabilities acquired showed the real opportunities that were needed to assist students with what they are able to do and have reason to value (Nussbaum 2000; Sen 1999).

Of equal significance, however, are the participants’ experiences of some elements of exclusionary practices. These experiences were exemplified by the social and academic suppression students experienced at the hands of the institution and from other students, some restrictions to making friends, being barred from interacting, negative stereotyping, labelling and discrimination. The concern for some students was that whilst they appreciated the skills acquired, they felt that their voices were not heard. From a Capability Approach analysis, students’ agency freedom was trampled upon. Agency is associated with the capability to make choices. Therefore, students would feel included if they were given the opportunity to choose what they want to do and that which they have reasons to value. Experiencing exclusionary practices means that some capabilities such as control over one’s environment (Nussbaum 2000), emotional integrity (Walker 2006); respect, dignity and recognition (Walker 2006) and physical activities (Terzi 2007) were not expanded. The formal access to an institution, where it does not also translate to access to education as a capability, is a restriction to functioning and hinders the development of other freedoms. This means that TVET institutions should create learning environments that respect students’ voices and support their freedoms to enable them to achieve their potential. The expectation is that higher education exposes all students to opportunities that promote their integrity as people who matter in society.

The experiences of the students highlight how the college environment created both inclusionary and exclusionary practices towards them. Exclusionary practices result in a lack of emotional integrity, which can affect other domains of student development. The effects of a disabling environment are that in terms of measuring the happiness level of the students in TVET, they were not in a happy environment. From a Capability Approach perspective, the scenario indicated that the student’s well-being achievement was limited. Ultimately, this hindered their capability to be educated. As argued by Unterhalter (2003), schooling, social opportunities and the development of reasoning are instrumental in capability development, and when people have those equal opportunities, they are bound to have a fair share of human agency.

In the final analysis, the findings have highlighted some of the challenges faced by TVET in realising the goal of inclusion of students with disabilities and demonstrate that TVET is struggling with the implementation and practice of inclusive education. The following are the recommendations to consider in creating opportunities for students to have reasons to value their beings and doings.

## Recommendations

Given the above discussion, we recommend the following:

- There seems to be a gap between the understanding of inclusion as indicated in policy and the actual practice of inclusion in education in Botswana’s TVET system. We recommend that policymakers should engage with stakeholders who implement policy. This would support Botswana to have an identified philosophy and paradigm common to both policymakers and implementers to operationalise the implementation and practice of Inclusive Education.
- We recommend continued institutional evaluation and engagement with developing inclusive strategies that can work within their context such as reconciling learning with the values of inclusive education rather than traditional special education. For example, having lecturers take surveys and keep journals to identify what has or has not worked for the institution would foster increased awareness. Developing a culture of responsiveness to individual student needs would support lecturers to select inclusive strategies suited to actual needs rather than offering preconceived notions of special education support.
- Institutional culture shifts in the way students with disabilities are viewed so that empowering strategies such as developing students’ agency, and freedom may be used to curb pigeonholing and labelling. This can be achieved if students with disabilities are given more opportunities to become active agents and participate socially, politically and educationally rather than limiting opportunities based on stereotypes of ability and capability.
- The educational experiences of students in these institutions should be a source of what TVET needs to consider particularly in identifying what can be aligned to the human development approach. Practically, this can be attained by providing more opportunities that improve students’ quality of life by improving TVET central spaces through focus on agency development for students with disabilities. This would support creating a favourable environment at institutional level and may be a first step in enabling policy ideals for inclusive TVET to be realised in practice.
- It is also clear from the study that lecturers and students without disabilities were instrumental in the success or challenges of inclusionary and exclusionary practices. It is recommended that the institution and department should support lecturers and students without disabilities to change their mindset about inclusion, where this is exclusionary and draw on the support of those lecturers and students without disability who already demonstrate inclusionary practices. For example, facilitating inductions and workshops about disability issues before students with disabilities arrive in college and also as an ongoing staff and student development throughout the academic year so that they become a support system for students with disabilities and instrumental stakeholders in implementing the inclusive education agenda.

## Conclusion

This article has reported on 14 in-depth interviews with students with mild intellectual disabilities about their experiences. Drawing from their interpreted experiences, the interviews revealed a parallel occurrence of both inclusion and exclusion indicative of an institution indistinct about policy implementation. The Capability Approach used helped us unpack the social arrangements that supported inclusionary practices in the institution and that, therefore, promoted capabilities. The capabilities that inclusionary practices promoted included learning disposition; practical reasoning; respect, dignity and recognition; social recognition and development of social networks. Inclusionary practices, therefore, afforded experiences of, amongst others, affiliation, social and epistemological inclusion. In contrast, the approach also revealed undeveloped capabilities such as voice, control of one’s environment, emotional integrity and physical activities associated with being excluded. Therefore, parallel to the experience of inclusion was the experience of exclusionary practices such as marginalisation, labelling, oppression and stigmatisation. Capabilities are simply opportunities to choose and therefore if students with disabilities have limited opportunities to choose, exclusionary practices are perpetuated, agency and freedom undermined and functioning underdeveloped. Failure to create and develop capabilities is a form of injustice and inequality, which is an exclusionary practice and undermines the realisation of inclusive ideals in TVET.

The picture that emerges is that the TVET institution under study appears to be struggling with the implementation of Inclusive Education. It is recommended that TVET should take note of the exclusionary practices identified and work towards equalising resources and capabilities whilst continuing to build on the observed inclusionary practices. In this way, students with disabilities may have improved opportunities and abilities to value what they have reason to be and do and ultimately have their well-being achievement realised.
